# Genetics of Chronic Kidney Disease Stages Across Ancestries: The PAGE Study

**DOI:** 10.3389/fgene.2019.00494

**Published:** 2019-05-24

**Authors:** Bridget M. Lin, Girish N. Nadkarni, Ran Tao, Mariaelisa Graff, Myriam Fornage, Steven Buyske, Tara C. Matise, Heather M. Highland, Lynne R. Wilkens, Christopher S. Carlson, S. Lani Park, V. Wendy Setiawan, Jose Luis Ambite, Gerardo Heiss, Eric Boerwinkle, Dan-Yu Lin, Andrew P. Morris, Ruth J. F. Loos, Charles Kooperberg, Kari E. North, Christina L. Wassel, Nora Franceschini

**Affiliations:** ^1^Department of Biostatistics, Gillings School of Global Public Health, University of North Carolina at Chapel Hill, Chapel Hill, NC, United States; ^2^Charles Bronfman Institute for Personalized Medicine, Icahn School of Medicine at Mount Sinai, New York, NY, United States; ^3^Division of Nephrology, Department of Medicine, Icahn School of Medicine at Mount Sinai, New York, NY, United States; ^4^Department of Biostatistics, Vanderbilt University Medical Center, Nashville, TN, United States; ^5^Vanderbilt Genetics Institute, Vanderbilt University Medical Center, Nashville, TN, United States; ^6^Department of Epidemiology, Gillings School of Global Public Health, University of North Carolina at Chapel Hill, Chapel Hill, NC, United States; ^7^The University of Texas Health Science Center at Houston, Houston, TX, United States; ^8^Department of Genetics, Rutgers University, Piscataway, NJ, United States; ^9^Epidemiology Program, University of Hawaii Cancer Center, Honolulu, HI, United States; ^10^Division of Public Health Sciences, Fred Hutchinson Cancer Research Center, Seattle, WA, United States; ^11^Department of Epidemiology, University of Washington, Seattle, WA, United States; ^12^Department of Preventive Medicine, University of Southern California, Los Angeles, CA, United States; ^13^Information Sciences Institute, University of Southern California, Marina del Rey, CA, United States; ^14^Department of Biostatistics, University of Liverpool, Liverpool, United Kingdom; ^15^Wellcome Centre for Human Genetics, University of Oxford, Oxford, United Kingdom; ^16^Mindich Child Health and Development Institute, Icahn School of Medicine at Mount Sinai, New York, NY, United States; ^17^Applied Sciences, Premier, Inc., Charlotte, NC, United States

**Keywords:** genetics, chronic kidney disease stages, genome-wide association studies, *APOL1*, end stage kidney disease, diverse populations, single nucleotide polymorphisms

## Abstract

**Background:**

Chronic kidney disease (CKD) is common and disproportionally burdens United States ethnic minorities. Its genetic determinants may differ by disease severity and clinical stages. To uncover genetic factors associated CKD severity among high-risk ethnic groups, we performed genome-wide association studies (GWAS) in diverse populations within the Population Architecture using Genomics and Epidemiology (PAGE) study.

**Methods:**

We assembled multi-ethnic genome-wide imputed data on CKD non-overlapping cases [4,150 mild to moderate CKD, 1,105 end-stage kidney disease (ESKD)] and non-CKD controls for up to 41,041 PAGE participants (African Americans, Hispanics/Latinos, East Asian, Native Hawaiian, and American Indians). We implemented a generalized estimating equation approach for GWAS using ancestry combined data while adjusting for age, sex, principal components, study, and ethnicity.

**Results:**

The GWAS identified a novel genome-wide associated locus for mild to moderate CKD nearby *NMT2* (rs10906850, *p* = 3.7 × 10^-8^) that replicated in the United Kingdom Biobank white British (*p* = 0.008). Several variants at the *APOL1* locus were associated with ESKD including the *APOL1* G1 rs73885319 (*p* = 1.2 × 10^-9^). There was no overlap among associated loci for CKD and ESKD traits, even at the previously reported *APOL1* locus (*p* = 0.76 for CKD). Several additional loci were associated with CKD or ESKD at *p*-values below the genome-wide threshold. These loci were often driven by variants more common in non-European ancestry.

**Conclusion:**

Our genetic study identified a novel association at *NMT2* for CKD and showed for the first time strong associations of the *APOL1* variants with ESKD across multi-ethnic populations. Our findings suggest differences in genetic effects across CKD severity and provide information for study design of genetic studies of CKD in diverse populations.

## Introduction

Chronic kidney disease (CKD) affects 15% of United States. adults and is a leading cause of death globally (Global Burden of Disease 2016 Causes of Death Collaborators, 2017). CKD is classified based on its causes, kidney function (estimated glomerular filtration rate, eGFR), and markers of kidney damage (Levin and Stevens, 2014). The risk of adverse outcomes and disability greatly increases in advanced CKD (Go et al., 2004; Saran et al., 2018). There is a high burden of CKD in non-European ancestry groups, including African Americans and Hispanics/Latinos (Collins et al., 2011). Genetic susceptibility explains in part ethnic differences in the burden of CKD, as illustrated by the African-ancestry *APOL1* G1 and G2 genotypes that contribute to increased CKD risk in individuals with African ancestry (Genovese et al., 2010; Kramer et al., 2017). Approximately 13% of African Americans carry two *APOL1* risk genotypes G1 (composed of two missense variants), G2 (a 6-base pair in-frame deletion) or are compound heterozygous of G1 and G2 genotypes. *APOL1* encodes an HDL cholesterol-binding protein but mechanisms related to CKD risk are unknown.

Few genome-wide association studies (GWAS) have been published for CKD as the primary outcome. These include studies of CKD progression such as the Chronic Renal Insufficiency Cohort Study (Parsa et al., 2017), and cause-specific CKD such as GWAS consortia that compared individuals with diabetic nephropathy with non-CKD diabetes controls (Iyengar et al., 2015; van Zuydam et al., 2018), in addition to studies of glomerular diseases (for example, IgA nephropathy, membranous nephropathy) (GWAS Catalog, 2019). CKD is a heterogeneous condition and its genetic determinants may vary by CKD severity, with more advanced CKD possibly reflecting stronger genetic risk. The genetic determinants of CKD severity have not been previously studied, particularly among individuals of diverse ancestries that vary in their genetic susceptibility.

Our recent research in diverse populations within the Continental Origins and Genetic Epidemiology Network (COGENT) Kidney Consortium identified 93 novel loci for eGFR, displaying homogenous effects across four major ancestries (Morris et al., 2019). Using Mendelian Randomization, we have shown that identified single nucleotide variants (SNVs) were causally related to a clinical diagnosis of CKD from International Classification of Disease (ICD) diagnosis billing codes in the United Kingdom Biobank. These SNVs, identified from the *trans-*ethnic analyses and showing homogenous effects across ancestries, more likely capture CKD genetic risk across diverse populations.

To identify novel risk loci associated with CKD severity (stages), we assembled multi-ethnic data on cases (4,150 mild to moderate CKD and 1,105 ESKD) and non-CKD controls for samples up to 41,041 participants within the Population Architecture using Genomics and Epidemiology (PAGE) study (Bien et al., 2016). We also examined the association of eGFR-identified GWAS variants with CKD stages using genetic variants reported by the COGENT-Kidney consortium.

## Materials and Methods

### PAGE Study Description

The PAGE consortium includes eligible minority participants from four studies. The Women’s Health Initiative (WHI) is a long-term, prospective, multi-center, and multi-ethnic cohort study investigating post-menopausal women’s health recruited from 1993 to 1998 at 40 centers across the United States (Anderson et al., 2003). WHI participants of European descent were excluded from analyses. The Hispanic Community Health Study/Study of Latinos (HCHS/SOL) is a multi-center study of Hispanic/Latinos with the goal of determining the role of acculturation in the prevalence and development of diseases relevant to Hispanic/Latino health. Starting in 2006, household sampling was used to recruit self-identified Hispanic/Latinos from four sites in San Diego, CA, Chicago, IL, Bronx, NY, and Miami, FL (Sorlie et al., 2010). The Multiethnic Cohort (MEC) is a population-based prospective cohort study recruiting men and women aged 45–75 from Hawaii and Los Angeles, California, in 1993–1996, that examines lifestyle risk factors and genetic susceptibility for cancer across five racial/ethnic groups (Kolonel et al., 2000). Only the African American, Japanese American, and Native Hawaiian participants for MEC were included in analyses. The BioMe BioBank is an Electronic Medical Record-linked biobank that integrates research data and clinical care information for consented patients at the Mount Sinai Medical Center, which serves diverse local communities of upper Manhattan with broad health disparities. Recruitment began in 2007 and continues at 30 clinical care sites throughout New York City. BioMe participants were African American, Hispanic/Latino, primarily of Caribbean origin (36%), Caucasian (30%), and Others who did not identify with any of the available options (9%) (Nadkarni et al., 2014). All PAGE participants have provided informed consent. Up to 41,041 participants with kidney phenotype information were included in analyses.

### Genotypes and Imputation

The genotyping and quality control (QC) in PAGE has been previously described (Bien et al., 2016). Briefly, 53,426 samples were genotyped centrally at the Center for Inherited Disease Research (CIDR) in the Johns Hopkins University using the Multi-Ethnic Genotyping Array (MEGA), Consortium version, consisting of 1,705,969 single nucleotide variants (SNV). Genotypes were called using the GenomeStudio version 2001.1, Genotyping Module 1.9.4, and GenTrain version 1.0. Extensive QC was performed to the combined genotyping data, which included checks for gender discrepancies, Mendelian inconsistencies, unexpected duplication, unexpected non-duplication, poor performance, or DNA mixture. Samples with identity issues, restricted consent, and duplicates were also removed (final sample 51,520 subjects). SNVs were filtered if they had a missing call rate ≥ 2%, more than 6 discordant calls in 988 study duplicates, > 1 Mendelian errors in 282 trios and 1,439 duos, a Hardy–Weinberg *p*-value < 10^-4^, sex difference in allele frequency ≥ 0.2 and sex difference in heterozygosity >0.3 for autosome chromosomes. After SNV QC, a total of 1,438,399 SNVs were available for analyses.

Imputation was done centrally at the University of Washington in combined samples. The study samples were phased with SHAPEIT2 (Delaneau et al., 2013) and imputed with IMPUTE2 (Howie et al., 2009) to the 1000 Genomes Project Phase 3 data release. Reference panel variants were restricted to a minor allele count (MAC) ≥2 across all 1000. Kinship coefficients were estimated using PC-Relate (Conomos et al., 2016). Principal components (PCs) were estimated in unrelated individuals within the global study population using SNVRelate package (Zheng et al., 2012). The first 10 PCs explained most of the genetic variation in the PAGE study population.

### CKD Phenotypes

For studies with available serum creatinine (HCHS/SOL, WHI), we calculated eGFR using the CKD-EPI equation and baseline cohort data (Inker et al., 2012). Mild to moderate CKD (referred as CKD) was defined by an eGFR between 15 and 60 ml/min/1.73 m^2^ (HCHS/SOL, WHI) or by an ICD-9 or 10 code in medical claims (585.1-585.5, 585.9, N18.1-N18.5, N18.9) (MEC, BioMe) (Nadkarni et al., 2014). Advanced CKD (referred as ESKD) was defined by an eGFR < 15 ml/min/1.73 m^2^ (HCHS/SOL), an ICD-9 or 10 code of 585.6 or N18.6 related to ESKD (MEC) or ESKD obtained through linkage to the United States Renal Data System (BioMe). CKD and ESKD cases were mutually exclusive. Controls were individuals with an eGFR > 60 ml/min/1.73 m^2^ or without ICD codes related to CKD or ESKD. In sensitivity analyses, we used two additional definitions for mild to moderate CKD: one based on ICD codes (MEC and BioMe data) and one based on eGFR from cohort studies (HCHS/SOL, WHI).

### Statistical Analyses

We performed genome-wide association analyses of the combined data using the software SUGEN, which implements a generalized estimating equation approach and empirically estimates within-family correlations without modeling the correlation structures of complex pedigrees (Lin et al., 2014). SUGEN adopts a modified version of the sandwich variance estimator, which replaces the empirical covariance matrix of the score vectors by the Fisher information matrix for unrelated subjects. We used logistic regression to analyze categorical phenotypes, and included age, sex, ten PCs, study, center (if available), and ethnicity as covariates. We filtered variants with an effective number <50 based on minor allele frequency of cases (MAF), number of participants (N) and imputation score from impute2 (info) using the following calculation [2^∗^MAF^∗^(1-MAF)^∗^N^∗^info] where N = total sample for a given phenotype. *P*-values were generated by score tests. Significant threshold for GWAS was *p* < 5.0 × 10^-8^ and suggestive threshold was a *p* < 1.0 × 10^-7^.

For SNVs with *p* < 10^-7^, we used the clumping procedure INDEP in Easystrata to identify independent signals at each locus which included on 1 Mb genomic interval flanking the lead SNVs. We also examined if the published loci for eGFR were associated with CKD or ESKD. We prioritized eGFR variants identified in the multi-ethnic COGENT-Kidney consortium and also used variants available in the Genome catalog for CKD and ESKD. For the *APOL1* locus, we performed analysis conditioning on the most significant SNV in the region. In sensitivity analysis, we assessed the significance of SNVs identified in the GWAS for CKD using CKD definition based on ICD codes or eGFR thresholds.

### Associations in the United Kingdom Biobank

We assessed the association of our identified variants in the United Kingdom Biobank for SNVs available in European ancestry listed in Tables 2, 3. We extracted *p*-values from United Kingdom Biobank using GeneATLAS (2019) for ICD-10 diagnosis codes N18 (chronic renal failure, 4,905 cases, and 447,359 controls), N19 (unspecified renal failure including uremia, kidney failure, azotemia, 1,516 cases and 450,748 controls) and renal/kidney failure (759 cases and 451,505 controls) (Gene ATLAS). Publicly available replication samples for CKD/ESKD in non-European ancestry were not available.

## Results

### Participant Characteristics

Overall, 41,041 individuals contributed data for CKD analyses (10.1% cases) and 31,694 individuals to ESKD analyses (3.4% cases) with non-overlapping cases. Cases were older and had a lower proportion of women compared to controls, and more comorbidities (Table 1). The study-specific contribution for cases and controls is shown in Supplementary Table 1.

**Table 1:** Descriptive characteristics of mild to moderate CKD and advance CKD stages.

Characteristics	CKD (mild to moderate)	Control	ESKD	Control
Total number	4,150	41,041	1,105	31,694
Mean age (SD), years	63.1 (9.5)	52.5 (13.1)	57.9 (11.4)	50.6 (13.8)
Female, %	61.2	68.2	45.9	59.4
Diabetes, %	53.3	24.7	56.1	24.1
Hypertension, %	92.5	49.6	98.7	48.0
African American, N (%)	1,532 (37)	13,761 (34)	584 (53)	8,429 (27)
Hispanic/Latino, N (%)	1,336 (32)	19,317 (47)	282 (26)	15,852 (50)
East Asian, N (%)	575 (14)	3,540 (9	112 (10)	3,536 (11)
Native Hawaiian, N (%)	608 (15)	2,892 (7)	96 (9)	2,892 (9)
American Indian, N (%)	42 (1)	595 (2)	1 (0)	49 (0)
Other ethnicity, N (%)	57 (1)	936 (2)	30 (3)	936 (3)


*SD, standard deviation; N, number.*

### Multi-Ethnic GWAS Findings

**FIGURE 1:**
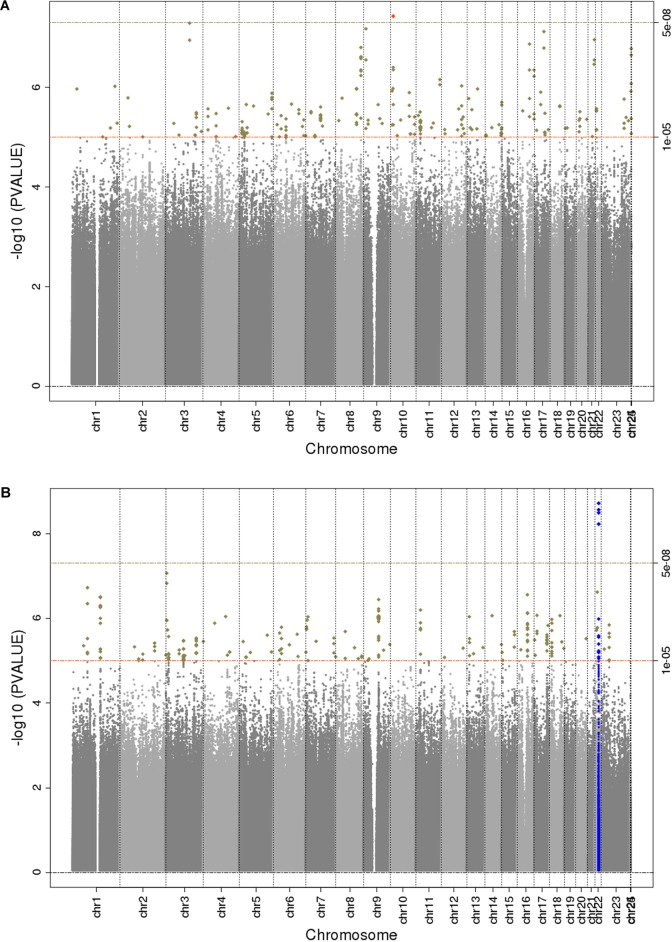
Manhattan plots for *trans-*ethnic GWAS of CKD **(A)** and ESKD **(B)**. Significant novel (red) and known (blue) loci are highlighted.

**FIGURE 2:**
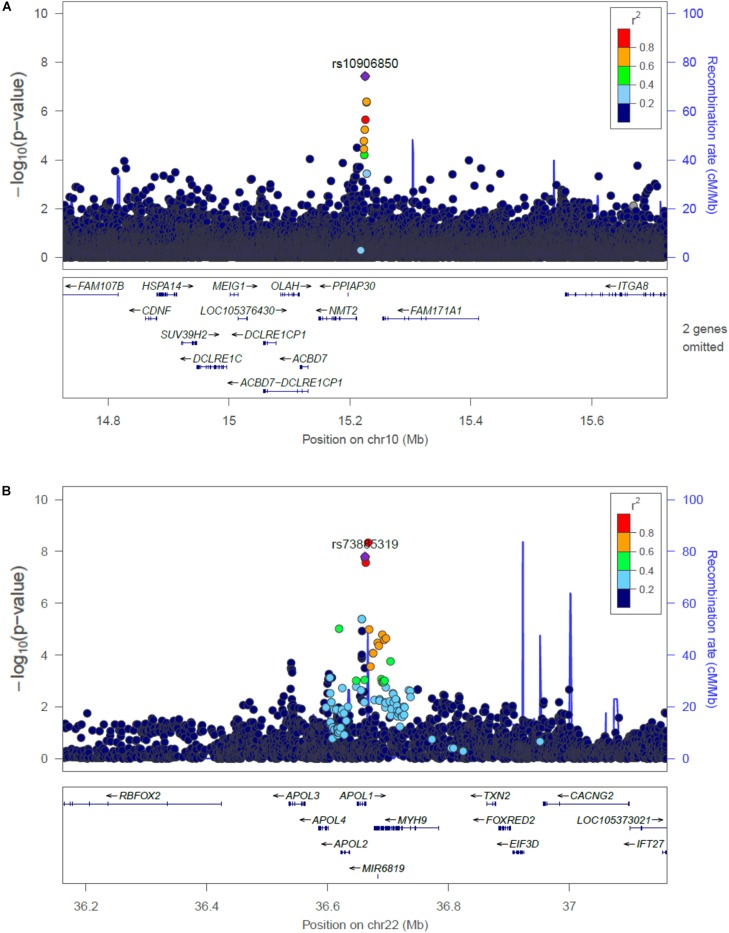
Regional plots of the association at the *NMT2* **(A)** with CKD, and the *APOL1* locus associated with ESKD **(B)**. Linkage disequilibrium was estimated from the PAGE multi-ethnic data.

The main findings from the GWAS of CKD and ESKD in combined multi-ethnic samples are shown in Tables 2, 3. Manhattan plots are shown in Figure 1 and quantile-quantile (QQ) are shown in Supplementary Figure 1. The genomic control lambdas were 1.025 for CKD and 1.026 for ESKD. These analyses identified two genome-wide associated loci: a chromosome 10 locus nearby *NMT2* associated with CKD (rs10906850, allele frequency 0.23, *p* = 3.7 × 10^-8^) (Table 2 and Figure 2A) and the chromosome 22 *APOL1* locus associated with ESKD (four common SNVs, including the two highly correlated *APOL1* G1 missense variants rs73885319 and rs60910145) (Figure 2B and Supplementary Table 2). *APOL1* G2 indel was not available in our data. Conditional analysis on the most significant SNV supported an independent association at the *APOL1* locus.

**Table 2:** Main findings for association with mild to moderate CKD at *p* < 10^-7^.

Chr:position (hg19)	SNV	Alleles	Allele frequency PAGE	Allele frequency 1000 genome project			Odds ratio	95% confidence interval	*p*	Nearby gene	Function or location
									
			Multi-ethnic	AFR	AMR	EUR					
1:226523031	rs76064236	G/T	0.005	NA	0.001	0.002	2.28	1.69, 3.16	9.5 × 10^-7^	*PARP1*	intergenic
3:128388015	rs146639727	C/G	0.004	0.013	NA	NA	3.05	2.04, 4.56	5.1 × 10^-8^	*RPN1*	intergenic
8:131963415	rs138873021	T/TAAG	0.629	0.260	0.752	0.818	1.17	1.11, 1.25	1.6 × 10^-7^	*ADCY8*	intron
9:12141349	rs186208070	A/T	0.009	0.020	0.003	0.001	2.07	1.59, 2.70	6.7 × 10^-8^	*TYRP1*	intergenic
10:15225054	rs10906850	C/T	0.225	0.109	0.242	0.325	1.18	1.11, 1.25	3.7 × 10^-8^	*NMT2*	intergenic
11:125840145	rs183951714	T/C	0.011	0.000	0.000	0.000	1.66	1.36, 2.02	7.0 × 10^-7^	*CDON*	intron
12:105784903	rs117329947	C/A	0.014	0.001	0.027	0.027	1.79	1.42, 2.25	9.3 × 10^-7^	*C12orf75*	intergenic
16:63000290	rs11645800	G/A	0.184	0.404	0.085	0.109	1.20	1.12, 1.28	1.4 × 10^-7^	*CDH8*	intergenic
16:86761390	rs147084429	T/C	0.019	0.050	0.001	NA	0.63	0.53, 0.76	4.5 × 10^-7^	*FOXL1*	intergenic
17:48825516	rs144210385	A/G	0.006	0.001	0.009	0.021	2.85	1.94, 4.17	7.6 × 10^-8^	*LUC7L3*	intron
21:41667378	rs2837554	G/A	0.701	NA	0.001	0.002	1.16	1.10, 1.24	3.1 × 10^-7^	*DSCAM*	intron
21:43048485	rs187652497	C/T	0.007	0.017	0.001	NA	2.23	1.66, 3.00	1.1 × 10^-7^	*LINC00111*	intergenic


*Chr, chromosome; SE, standard error; SNV, single nucleotide variant; NA, not available; AFR, African; EUR, European; AMR, Ad Mixed American. Models adjusted for age, sex, study, race/ethnicity and PCs.*

**Table 3:** Main association findings with ESKD at *p* < 10^-7^.

Chr:position (hg19)	SNV	Alleles	Allele Freq PAGE	Allele Frequency 1000 Genome Project			Odds Ratio	95% Confidence Interval	*p*	Nearby gene	Function or location
									
			Multi-ethnic	AFR	AMR	EUR					
1:78634021	rs77138376	A/G	0.009	NA	NA	NA	2.63	1.83, 3,78	1.9 × 10^-7^	*GIPC2*	intergenic
1: 146810798	rs12032578	C/T	0.088	0.160	0.033	0.007	1.44	1.25, 1.66	3.1 × 10^-7^	*LINC00624*	intergenic
3: 5547449	rs112407915	A/T	0.020	0.047	0.003	NA	1.96	1.53, 2.51	8.6 × 10^-8^	*MIR4790*	intergenic
4: 118841271	rs114425659	A/G	0.039	0.023	0.063	0.080	1.87	1.45, 2.39	9.1 × 10^-7^	*NDST3*	intergenic
7:11617925	rs74614630	C/T	0.012	0.030	0.003	NA	2.25	1.63, 3.11	9.2 × 10^-7^	*THSD7A*	intron
9: 79608946	rs115747230	C/T	0.021	0.061	0.004	0.001	1.88	1.47, 2.39	3.6 × 10^-7^	*FOXB2*	intergenic
11:24231483	rs116510623	G/A	0.034	0.107	0.009	0.001	1.65	1.35,2.01	6.4 × 10^-7^	*LUZP2*	intergenic
13:32883196	rs60236946	T/C	0.021	0.071	0.006	NA	1.86	1.45, 2.38	9.2 × 10^-7^	*ZAR1L*	intron
14:56825071	rs115007604	A/G	0.008	0.023	0.006	NA	2.67	1.81, 3.95	8.7 × 10^-7^	*PELI2*	intergenic
16: 53886224	rs7189997	G/A	0.009	0.027	0.003	NA	2.44	1.73, 3.42	2.8 × 10^-7^	*FTO*	intron
16: 54413730	rs8050506	A/G	0.211	0.324	0.108	0.119	1.29	1.17, 1.43	7.5 × 10^-7^	*IRX3*	5’-UTR
17:14189909	rs191540116	A/G	0.011	0.026	0.01	0.000	2.32	1.66, 3.25	8.6 × 10^-7^	*HS3ST3B1*	intergenic
18: 54764414	rs12963285	C/T	0.166	0.269	0.170	0.038	0.74	0.66, 0.84	9.7 × 10^-7^	*LINC-ROR*	5’-UTR
22:29461285	rs138572244	A/G	0.008	0.022	0.001	NA	2.90	1.94, 4.35	2.4 × 10^-7^	*C22orf31*	intergenic
22: 36661906	rs73885319	G/A	0.077	0.260	0.009	NA	1.51	1.32, 1.72	1.9 × 10^-9^	*APOL1*	missense


*Chr, chromosome; SE, standard error; SNV, single nucleotide polymorphism; NA, not available; AFR, African; EUR, European; AMR, Ad Mixed American. Models adjusted for age, sex, study, race/ethnicity and PCs.*

Several loci with low frequency SNVs had suggestive evidence for association including SNVs located nearby *RPN1* (*p* = 5.1 × 10^-8^), *TYRP1* (*p* = 6.7 × 10^-8^), and *LUC7L3* (*p* = 7.6 × 10^-8^) associated with CKD, and *MIR4790* (*p* = 8.6 × 10^-8^) associated with ESKD. Except for *LUC7L3*, the most significant SNV at these loci was rare or not present in European ancestry. For example, the *TYRP1* variant allele frequency in 1000 Genomes Project is 0.02 in African ancestry and 0.001 in European ancestry, and *RPN1* and *MIR4790* SNVs are not available in 1000 Genomes Project European ancestry samples. Additional SNVs associated with ESKD at *p* < 10^-7^ included a low frequency intronic variant in *FTO* (rs7189997, *p* = 2.8 -10^-7^) and a common variant nearby *IRX3* (rs8050506, *p* = 9.94 × 10^-7^). Both SNVs were also more common in African ancestry reference panels (Table 3).

Nine of the SNVs with significant or suggestive association with CKD or ESKD (listed in Tables 2, 3) were available for replication in the United Kingdom Biobank white British samples. The SNV rs10906850 nearby *NMT2* was significantly associated with ICD code for renal/kidney failure (*p* = 0.008) and rs11645800 nearby *CDH8* was nominally associated with renal/kidney failure (Table 4). The common indel rs138873021 was not available in the United Kingdom Biobank.

**Table 4:** Association of SNVs identified for CKD or ESKD in white British in the United Kingdom Biobank for three CKD diagnosis.

SNV	Coded allele	Minor Allele frequency United Kingdom Biobank	N18 chronic renal failure^∗^		N19 unspecified renal failure^∗^		Renal/kidney failure^∗^		PAGE discovery samples
						
			*p*	OR	*p*	OR	*p*	OR	OR
**CKD SNVs**									
rs10906850	C	0.37	0.16	1.03	0.83	1.01	0.0089	1.15	1.18
rs117329947	C	0.03	0.75	1.02	0.60	0.95	0.34	1.02	1.79
rs11645800	G	0.35	0.63	1.01	0.33	1.04	0.028	1.13	1.20
rs144210385	A	0.02	0.94	1.01	0.31	0.86	0.69	0.92	2.85
rs2837554	G	0.08	0.57	1.02	0.79	0.98	0.12	0.86	1.16
**ESKD SNVs**									
rs12032578	C	0.003	0.96	1.01	0.17	0.61	0.34	0.62	1.44
rs114425659	A	0.05	0.32	0.96	0.87	0.99	0.12	0.83	1.87
rs8050506	A	0.11	0.35	1.03	0.84	1.01	0.64	0.96	1.29
rs12963285	C	0.04	0.34	0.95	0.02	0.80	0.35	0.88	0.74


*Indels rs61555057 and rs138873021 are not available in the United Kingdom Biobank. SNV, single nucleotide variant; OR, odds ratio for association of coded allele. ^∗^ICD-10 codes in the United Kingdom Biobank.*

### Cross-Association of Significant SNVs for CKD and ESKD and Sensitivity Analyses

At the *NMT2* locus associated for CKD, the most significant SNV was not associated with ESKD (*p* = 0.82). At the *APOL1* locus associated with ESKD, rs73885319 (and other variants) were not associated with CKD (*p* = 0.76). To explore heterogeneity in the definition for CKD that could explain our findings, we examined the association of the genome-wide associated significant SNVs in samples stratified by CKD definition based on ICD code (*n* = 4,698 cases, *n* = 18,764 controls) or eGFR thresholds (*n* = 3,179 cases, *n* = 18,550 controls). The *NMT2* SNV rs10906850 was associated with CKD using both definitions (*p* = 2.4 × 10^-6^ for ICD codes and *p* = 8.2 × 10^-3^ for eGFR thresholds) and there was consistency in direction of effects. Conversely, *APOL1* rs73885319 was not associated with CKD using either definition (*p* > 0.05).

### Association of Previously Reported eGFR SNVs From the COGENT-Kidney Consortium With CKD Stages in PAGE

Given PAGE studies included diverse (non-European) participants, we selected 93 eGFR SNVs identified in the *trans-*ethnic COGENT-Kidney Consortium to assess their association with CKD and ESKD in PAGE. Seventeen loci were associated with CKD and six loci were associated with ESKD at nominal *p*-values (*p* < 0.05). These SNVs had concordant effect estimates between the COGENT-Kidney eGFR lowering allele that showed increased odds of CKD or ESKD (Supplementary Table 3). The *PDILT/UMOD* was the only locus that was associated with both CKD and ESKD.

## Discussion

The main finding of this study is the identification of a new locus for mild to moderate CKD nearby *NMT2* for a SNV common across ancestries. The study also shows for the first time genome-wide associations of the *APOL1* SNVs with ESKD across multi-ethnic populations. Several other leading low frequency variants showed suggestive association with CKD traits. Most of the low frequency associated SNVs were more common in reference datasets of African ancestry and rare or absent in individuals of European ancestry, particularly for findings related to ESKD. These findings were driven by our discovery samples, which is composed of a large number of non-European ancestry including African Americans (34%) and Hispanics/Latinos (46%). Only nine SNVs were available in the United Kingdom Biobank for a sample of European ancestry. We replicated the association at the *NMT2* locus, which also showed consistent direction of effects for alleles between PAGE and the United Kingdom Biobank samples. Of the remaining eight SNVs brought for replication, four had consistent direction of effect between CKD discovery and the United Kingdom Biobank ICD code N18 replication, and one showed consistent direction of effect between ESKD discovery and United Kingdom Biobank ICD code N19 and renal/kidney failure replication, although *p*-values were not significant. We were unable to replicate other variants due to their low frequency or unavailability in samples of European ancestry, and lack of comparable publicly available summary results for CKD traits in non-European populations.

Single nucleotide variant rs10906850 is an intergenic variant located nearby *NMT2*, a gene that encodes a protein involved in regulating the function and localization of signaling proteins. The SNV is an expression quantitative trait (eQTL) for *NMT2* in tibial artery (*p* = 4.7 × 10^-9^), adipose tissue (*p* = 5.7 × 10^-8^) and skin (*p* = 1.6 × 10^-7^). This locus has not been previously associated with kidney traits. Additional locus identified at *p* < 10^-7^ for CKD includes *ADCY8*, which has been previously described in the CRIC study for association with eGFR decline among non-diabetic African Americans, although not at genome-wide significant level (Parsa et al., 2017). Our SNV in the region (rs138873021) is in linkage disequilibrium with the SNV identified in the CRIC study (rs4492355, *p* = 1.3 × 10^-7^ in the CRIC study, D’ = 0.90, *r*^2^ = 0.01 in 1000 Genomes Project African ancestry), although rs4492355 is not associated with CKD in the study (*p* = 0.53). Two loci associated with ESKD have been previously associated with obesity traits (*FTO* and *IRX3*), but our SNVs are low frequency and more common in African ancestry. Our findings related to the association of eGFR SNVs identified in the COGENT-Kidney consortium with CKD stages supports heterogeneity in genetic effects across CKD stages. We found a larger number of COGENT-Kidney eGFR lowering SNVs associated with increased CKD than eGFR lowering SNVs associated with increased ESKD. Only one locus was associated with both CKD and ESKD in PAGE: rs77924615 at the *PDILT/UMOD* locus, which showed nominal associations with these traits.

Chronic kidney disease is a heterogeneous disease in its etiology and clinical manifestation, with varying rates of progression to advanced stages. There is still little understanding on the mechanisms related to these varying patterns of disease severity even within the same disease etiology, for example, diabetic nephropathy. An interesting finding of our study is that there may be differences in genetic susceptibility based on the severity of the disease manifestation. We found little overlap of the most significant loci associated with the CKD phenotype (that includes mild to moderate CKD stages) and ESKD (which reflects advanced CKD). For example, the known *APOL1* G1 genotype was not associated with CKD. The *NMT2* SNV was significantly associated with CKD but not with ESKD. Although our study lacks information on the *APOL1* G2 SNV, we did not identify additional associations at the chromosome 22 locus in conditional analyses. The *APOL1* risk genotypes, including G1, were identified in admixture mapping for ESKD attributed to hypertension, FSGS or HIV (Genovese et al., 2010), and associations have been replicated in population studies although not at the genome-wide significant level (Foster et al., 2013; Kramer et al., 2017). Our study provides evidence for *APOL1* association with ESKD among diverse populations and for ESKD not selected for a specific disease etiology.

There are several possible explanations for the different genetic findings by CKD stages. Advanced CKD (ESKD) may have a stronger genetic component related to CKD progression, whereas mild to moderate CKD may capture more genetic factors related to CKD initiation. Mild to moderate CKD likely has less genetic influences due to inclusion of older individuals (aging process) and due to environmental factors. Alternatively, there is greater heterogeneity in our definition of mild to moderate CKD, opening the possibility of misclassified cases among individuals with an eGFR around the threshold of 60 ml/min/1.73 m^2^. However, our sensitivity analyses in CKD subgroups defined by ICD codes or eGFR thresholds did not show differences in the association for our significant *NMT2* locus. Overall, these findings provide important information for the study design of genetic studies for CKD, which should consider phenotype heterogeneity and severity of disease particularly when CKD is defined using ICD billing codes and when the definition includes a mixed case of CKD identified through biomarkers or clinical disease.

In conclusion, our multi-ethnic study identified a novel locus for mild to moderate CKD and replicated a known locus for ESKD. Our results highlight the need for more studies in diverse populations to identify genetic risk factors in populations at higher risk for CKD. It also underscores the current limitations of genetic research in these populations, including the lack of suitable replication samples for non-European ancestry variants.

## Ethics Statement

All human research was approved by the relevant institutional review boards and conducted according to the Declaration of Helsinki. All participants provided written informed consent.

## Author Contributions

NF, GN, and CW conceived and designed the experiments. NF and CW coordinated the project. JA, RT, MG, HMH, SB, TM, and D-YL performed the quality control of genotypes and phenotype data, or support for statistical methods. BL, NF, and CW performed the statistical analyses. BL, NF, LW, and RT drafted the manuscript. All authors critically revised the manuscript.

## Conflict of Interest Statement

The authors declare that the research was conducted in the absence of any commercial or financial relationships that could be construed as a potential conflict of interest.
