# Supplementary material for: Genetics of Chronic Kidney Disease Stages Across Ancestries: The PAGE Study
Source: Front Genet. 2019 May 24;10:494. doi: 10.3389/fgene.2019.00494 (PMC6544117; doi:10.3389/fgene.2019.00494)

SUPPLEMENTARY MATERIAL

**Supplementary Table 1**. Distribution of cases and controls within PAGE studies

| Study | CKD | Control | ESKD | Control |
| --- | --- | --- | --- | --- |
| BioME | 1,404 | 10,091 | 672 | 10,091 |
| MEC | 1,874 | 10,093 | 417 | 10,093 |
| SOL | 394 | 11,510 | 16 | 11,510 |
| WHI | 478 | 9,347 | 0 | 0 |
| Overall | 4,150 | 41,041 | 1,105 | 31,694 |

| CHR | POS (hg19) | SNV | Coded allele | Other allele | FREQ | OR | 95% CI | *p* | Function (AA change) |
| --- | --- | --- | --- | --- | --- | --- | --- | --- | --- |
| 22 | 36661906 | rs73885319 | G | A | 0.08 | 1.51 | 1.32, 1.72 | 1.9x10^-9^ | Missense (324G)* |
| 22 | 36662034 | rs60910145 | G | T | 0.08 | 1.50 | 1.31, 1.72 | 3.2x10^-9^ | Missense (I366M)* |
| 22 | 36663213 | rs58384577 | C | T | 0.08 | 1.49 | 1.30, 1.71 | 6.0x10^-9^ | UTR3 |
| 22 | 36667154 | rs60295735 | A | G | 0.08 | 1.52 | 1.33, 1.75 | 2.7x10^-9^ | Intergenic |

**Supplementary Table 2**. Four common SNPs at the *APOL1* locus (chromosome 22) associated with ESKD (N=32,799, N cases=1,105)

**APOL1* G1; OR, odds ratio; CI, confidence interval; AA, aminoacid

**Supplementary Table 3**. Association of eGFR lowering alleles of SNVs from the COGENT-Kidney Consortium with PAGE CKD and ESKD traits. Note that an OR>1.0 for CKD/ESKD is concordant with lowering effects on eGFR SNV alleles. Highlighted in red/ bold, significant association at p-value<0.05 and with concordant direction of effect. Note that an odds ratio (OR) > 1.0 for CKD/ESKD is considered concordant direction of effects for an eGFR decreasing coded allele. SNVs rs117463603, rs142647267 and rs187355703 were not available

|  |  |  | Coded | Other | Frequency | CKD | | | | ESKD | | | |
| --- | --- | --- | --- | --- | --- | --- | --- | --- | --- | --- | --- | --- | --- |
| SNV | **Chr** | **Pos (hg19)** | **Allele** | **Allele** | **Coded Allele** | **OR** | **Number** | **Cases** | **Pvalue** | **OR** | **Number** | **Cases** | **Pvalue** |
| rs45619934 | 1 | 15911947 | T | G | 0.24 | 1.00 | 45191 | 4150 | 0.89617 | 0.97 | 32799 | 1105 | 0.62379 |
| rs4525087 | 1 | 23692229 | C | A | 0.48 | 0.96 | 45191 | 4150 | 0.17021 | 0.96 | 32799 | 1105 | 0.37615 |
| rs12722725 | 1 | 113258681 | C | T | 0.04 | **1.15** | **45191** | **4150** | **0.03928** | 0.95 | 32799 | 1105 | 0.67596 |
| rs267738 | 1 | 150940625 | T | G | 0.92 | 1.05 | 45191 | 4150 | 0.35294 | 1.20 | 32799 | 1105 | 0.07511 |
| rs2070803 | 1 | 155157715 | A | G | 0.48 | 1.04 | 45191 | 4150 | 0.11679 | 1.09 | 32799 | 1105 | 0.05140 |
| rs2842870 | 1 | 156200671 | T | C | 0.57 | 1.03 | 45191 | 4150 | 0.22385 | 0.98 | 32799 | 1105 | 0.65136 |
| rs3850625 | 1 | 201016296 | G | A | 0.94 | 1.03 | 45191 | 4150 | 0.62567 | 0.88 | 32799 | 1105 | 0.19989 |
| rs10158537 | 1 | 243488186 | G | C | 0.23 | 1.05 | 45191 | 4150 | 0.11924 | 0.97 | 32799 | 1105 | 0.63266 |
| rs807603 | 2 | 15792518 | T | C | 0.41 | 1.00 | 45191 | 4150 | 0.98804 | 1.05 | 32799 | 1105 | 0.29654 |
| rs13417750 | 2 | 18681365 | A | G | 0.26 | 1.04 | 45191 | 4150 | 0.19935 | 1.08 | 32799 | 1105 | 0.13985 |
| rs1260326 | 2 | 27730940 | C | T | 0.71 | **1.06** | **45191** | **4150** | **0.04310** | 0.99 | 32799 | 1105 | 0.83360 |
| rs1527649 | 2 | 54581356 | C | T | 0.24 | 1.03 | 45191 | 4150 | 0.28473 | 0.98 | 32799 | 1105 | 0.68606 |
| rs6546869 | 2 | 73895765 | G | A | 0.74 | 1.06 | 45191 | 4150 | 0.06243 | **1.14** | **32799** | **1105** | **0.01777** |
| rs11123169 | 2 | 113967075 | C | T | 0.33 | 1.01 | 45191 | 4150 | 0.66313 | 1.05 | 32799 | 1105 | 0.32475 |
| rs13026220 | 2 | 148586459 | G | A | 0.29 | 0.98 | 45191 | 4150 | 0.42107 | 1.04 | 32799 | 1105 | 0.48408 |
| rs77335736 | 2 | 163089866 | C | T | 0.88 | 1.09 | 45191 | 4150 | 0.04761 | 1.01 | 32799 | 1105 | 0.89979 |
| rs3770636 | 2 | 170202833 | T | G | 0.91 | 1.06 | 45191 | 4150 | 0.15630 | 0.94 | 32799 | 1105 | 0.39660 |
| rs35955110 | 2 | 178143371 | C | T | 0.34 | **1.06** | **45191** | **4150** | **0.04150** | 1.04 | 32799 | 1105 | 0.40302 |
| rs1047891 | 2 | 211540507 | A | C | 0.30 | **1.10** | **45191** | **4150** | **0.00056** | 1.04 | 32799 | 1105 | 0.40638 |
| rs7587010 | 2 | 217674989 | T | G | 0.53 | 1.02 | 45191 | 4150 | 0.54903 | 1.02 | 32799 | 1105 | 0.71639 |
| rs36070911 | 3 | 38498439 | G | A | 0.45 | 1.03 | 45191 | 4150 | 0.26869 | 0.97 | 32799 | 1105 | 0.49128 |
| rs2250067 | 3 | 121644030 | T | C | 0.43 | 1.02 | 45191 | 4150 | 0.36150 | 0.96 | 32799 | 1105 | 0.34323 |
| rs1511299 | 3 | 141716072 | T | C | 0.84 | 1.06 | 45191 | 4150 | 0.09680 | 1.08 | 32799 | 1105 | 0.27288 |
| rs13081203 | 3 | 185322643 | A | G | 0.38 | 1.00 | 45191 | 4150 | 0.86054 | 1.09 | 32799 | 1105 | 0.07168 |
| rs13108218 | 4 | 3443931 | G | A | 0.53 | 1.04 | 45191 | 4150 | 0.09550 | 1.04 | 32799 | 1105 | 0.37765 |
| rs12509595 | 4 | 81182554 | T | C | 0.80 | **1.08** | **45191** | **4150** | **0.01345** | 1.04 | 32799 | 1105 | 0.55295 |
| rs223401 | 4 | 103738972 | T | C | 0.58 | 1.00 | 45191 | 4150 | 0.94631 | 0.98 | 32799 | 1105 | 0.58693 |
| rs10066990 | 5 | 34509825 | A | G | 0.22 | 1.05 | 45191 | 4150 | 0.08329 | 0.92 | 32799 | 1105 | 0.15145 |
| rs13179493 | 5 | 39426307 | C | T | 0.14 | 1.04 | 45191 | 4150 | 0.37017 | 0.92 | 32799 | 1105 | 0.28357 |
| rs7719168 | 5 | 53292390 | A | C | 0.94 | **1.12** | **45191** | **4150** | **0.04474** | 1.05 | 32799 | 1105 | 0.64265 |
| rs113246091 | 5 | 67739274 | A | G | 0.06 | 0.96 | 45191 | 4150 | 0.46586 | 1.05 | 32799 | 1105 | 0.58892 |
| rs3812036 | 5 | 176813404 | T | C | 0.16 | **1.08** | **45191** | **4150** | **0.02328** | 0.94 | 32799 | 1105 | 0.38623 |
| rs6935129 | 6 | 34371707 | A | G | 0.20 | 1.05 | 45191 | 4150 | 0.13038 | 0.99 | 32799 | 1105 | 0.88748 |
| rs881858 | 6 | 43806609 | A | G | 0.60 | 1.05 | 45191 | 4150 | 0.09536 | 0.95 | 32799 | 1105 | 0.27497 |
| rs9375818 | 6 | 131882078 | A | G | 0.33 | 0.99 | 45191 | 4150 | 0.79426 | 1.04 | 32799 | 1105 | 0.37141 |
| rs316020 | 6 | 160669081 | G | A | 0.90 | 1.08 | 45191 | 4150 | 0.05004 | 1.01 | 32799 | 1105 | 0.88745 |
| rs62435145 | 7 | 1286567 | T | G | 0.39 | 1.04 | 45191 | 4150 | 0.15147 | 1.01 | 32799 | 1105 | 0.82385 |
| rs856563 | 7 | 46723510 | C | T | 0.57 | 1.03 | 45191 | 4150 | 0.21402 | 0.98 | 32799 | 1105 | 0.67957 |
| rs848486 | 7 | 77552127 | G | A | 0.42 | 1.02 | 45191 | 4150 | 0.49497 | 0.97 | 32799 | 1105 | 0.46777 |
| rs10265221 | 7 | 151414329 | C | T | 0.18 | 1.06 | 45191 | 4150 | 0.07854 | 1.07 | 32799 | 1105 | 0.24066 |
| rs6971211 | 7 | 155664686 | T | C | 0.38 | 0.98 | 45191 | 4150 | 0.41421 | 1.00 | 32799 | 1105 | 0.93832 |
| rs7007761 | 8 | 23719571 | T | C | 0.31 | 1.00 | 45191 | 4150 | 0.87543 | 0.94 | 32799 | 1105 | 0.18906 |
| rs4489283 | 8 | 32399662 | T | C | 0.42 | 1.02 | 45191 | 4150 | 0.37630 | 1.07 | 32799 | 1105 | 0.15781 |
| rs2001945 | 8 | 126477978 | C | G | 0.62 | 0.98 | 45191 | 4150 | 0.36262 | 0.99 | 32799 | 1105 | 0.79773 |
| rs61237993 | 9 | 34130435 | G | A | 0.82 | 0.95 | 45191 | 4150 | 0.13577 | 0.96 | 32799 | 1105 | 0.59221 |
| rs2039424 | 9 | 71432174 | G | A | 0.33 | 1.01 | 45191 | 4150 | 0.58364 | 0.96 | 32799 | 1105 | 0.34971 |
| rs13283416 | 9 | 119301607 | G | T | 0.40 | 1.02 | 45191 | 4150 | 0.48812 | **0.91** | **32799** | **1105** | **0.03052** |
| rs80282103 | 10 | 899071 | T | A | 0.13 | **1.10** | **45191** | **4150** | **0.01436** | 1.08 | 32799 | 1105 | 0.23230 |
| rs7475348 | 10 | 69965177 | C | T | 0.67 | 1.03 | 45191 | 4150 | 0.26189 | 0.97 | 32799 | 1105 | 0.51234 |
| rs4418728 | 10 | 94839724 | T | G | 0.43 | 1.04 | 45191 | 4150 | 0.12900 | 1.00 | 32799 | 1105 | 0.97303 |
| rs6892 | 10 | 104575870 | A | G | 0.85 | 1.07 | 45191 | 4150 | 0.07238 | 0.92 | 32799 | 1105 | 0.30631 |
| rs4962691 | 10 | 126424137 | T | C | 0.41 | 1.00 | 45191 | 4150 | 0.94773 | 1.00 | 32799 | 1105 | 0.93085 |
| rs7482894 | 11 | 2121264 | T | C | 0.49 | 0.97 | 45191 | 4150 | 0.24896 | **1.13** | **32799** | **1105** | **0.01775** |
| rs963837 | 11 | 30749090 | T | C | 0.70 | **1.10** | **45191** | **4150** | **0.00148** | 1.01 | 32799 | 1105 | 0.82843 |
| rs11039221 | 11 | 47427739 | T | C | 0.27 | 1.04 | 45191 | 4150 | 0.13531 | **0.87** | **32799** | **1105** | **0.00815** |
| rs11604451 | 11 | 65551710 | T | C | 0.22 | 0.99 | 45191 | 4150 | 0.64953 | 0.97 | 32799 | 1105 | 0.57354 |
| rs2063724 | 11 | 78133077 | T | C | 0.69 | 1.00 | 45191 | 4150 | 0.95777 | 1.04 | 32799 | 1105 | 0.43866 |
| rs10774020 | 12 | 348876 | C | T | 0.44 | 0.97 | 45191 | 4150 | 0.24824 | 1.03 | 32799 | 1105 | 0.48279 |
| rs632887 | 12 | 3392351 | G | A | 0.44 | 0.98 | 45191 | 4150 | 0.51588 | 0.99 | 32799 | 1105 | 0.86345 |
| rs1275609 | 12 | 76271183 | G | A | 0.55 | 1.00 | 45191 | 4150 | 0.93708 | 1.02 | 32799 | 1105 | 0.63946 |
| rs79105258 | 12 | 111718231 | A | C | 0.03 | 1.02 | 45191 | 4150 | 0.72623 | 0.98 | 32799 | 1105 | 0.87605 |
| rs34445998 | 13 | 42757213 | C | T | 0.84 | 0.97 | 45191 | 4150 | 0.38510 | 1.01 | 32799 | 1105 | 0.86129 |
| rs584480 | 13 | 72345505 | C | T | 0.38 | 1.01 | 45191 | 4150 | 0.72647 | 0.97 | 32799 | 1105 | 0.46922 |
| rs9920185 | 15 | 39273575 | C | A | 0.70 | 1.05 | 45191 | 4150 | 0.06216 | 1.02 | 32799 | 1105 | 0.68339 |
| rs2486288 | 15 | 45712339 | C | T | 0.73 | 1.05 | 45191 | 4150 | 0.10467 | 0.98 | 32799 | 1105 | 0.72509 |
| rs62005941 | 15 | 53915766 | C | G | 0.73 | **1.07** | **45191** | **4150** | **0.02395** | 0.99 | 32799 | 1105 | 0.82743 |
| rs11636251 | 15 | 76239020 | T | C | 0.39 | **1.06** | **45191** | **4150** | **0.02292** | 0.99 | 32799 | 1105 | 0.91284 |
| rs62035088 | 15 | 81165842 | G | A | 0.86 | 1.07 | 45191 | 4150 | 0.09126 | 0.95 | 32799 | 1105 | 0.48749 |
| rs11858316 | 15 | 99249029 | C | T | 0.57 | 1.02 | 45191 | 4150 | 0.38084 | 0.99 | 32799 | 1105 | 0.87471 |
| rs77924615 | 16 | 20392332 | G | A | 0.85 | **1.11** | **45191** | **4150** | **0.00344** | **1.25** | **32799** | **1105** | **0.00181** |
| rs12935539 | 16 | 51754991 | C | T | 0.25 | 1.04 | 45191 | 4150 | 0.20434 | 1.05 | 32799 | 1105 | 0.35170 |
| rs9888796 | 16 | 68297589 | T | C | 0.17 | 1.01 | 45191 | 4150 | 0.68622 | 1.02 | 32799 | 1105 | 0.75569 |
| rs11641050 | 16 | 69622104 | C | T | 0.67 | 1.05 | 45191 | 4150 | 0.08083 | 0.98 | 32799 | 1105 | 0.61365 |
| rs2460449 | 16 | 89700747 | A | G | 0.25 | 1.01 | 45191 | 4150 | 0.79483 | 0.96 | 32799 | 1105 | 0.55872 |
| rs11871125 | 17 | 19432393 | T | C | 0.27 | **1.07** | **45191** | **4150** | **0.01580** | 0.96 | 32799 | 1105 | 0.46770 |
| rs4795384 | 17 | 37716771 | G | C | 0.65 | 1.03 | 45191 | 4150 | 0.31866 | 0.94 | 32799 | 1105 | 0.16644 |
| rs9895661 | 17 | 59456589 | C | T | 0.44 | 1.02 | 45191 | 4150 | 0.47389 | 0.98 | 32799 | 1105 | 0.72332 |
| rs1719934 | 18 | 5585158 | G | A | 0.32 | 1.02 | 45191 | 4150 | 0.38849 | **1.13** | **32799** | **1105** | **0.01826** |
| rs16942751 | 18 | 24393213 | A | C | 0.13 | 1.05 | 45191 | 4150 | 0.18573 | 0.91 | 32799 | 1105 | 0.17618 |
| rs2337106 | 18 | 46460903 | C | G | 0.34 | 1.02 | 45191 | 4150 | 0.53047 | 0.99 | 32799 | 1105 | 0.85895 |
| rs896642 | 18 | 59379074 | C | T | 0.59 | 1.00 | 45191 | 4150 | 0.92641 | 1.05 | 32799 | 1105 | 0.32287 |
| rs8096658 | 18 | 77156537 | G | C | 0.32 | **1.07** | **45191** | **4150** | **0.02453** | 1.03 | 32799 | 1105 | 0.58874 |
| rs8108623 | 19 | 18408519 | A | C | 0.65 | 1.03 | 45191 | 4150 | 0.34589 | 1.05 | 32799 | 1105 | 0.39760 |
| rs7252778 | 19 | 33360369 | A | C | 0.48 | 1.02 | 45191 | 4150 | 0.33409 | 0.98 | 32799 | 1105 | 0.60553 |
| rs2273684 | 20 | 33529766 | G | T | 0.47 | 0.99 | 45191 | 4150 | 0.59407 | 0.94 | 32799 | 1105 | 0.22984 |
| rs17216707 | 20 | 52732362 | T | C | 0.82 | **1.10** | **45191** | **4150** | **0.01320** | 1.02 | 32799 | 1105 | 0.80890 |
| rs1758206 | 20 | 62336334 | T | C | 0.05 | 1.00 | 45191 | 4150 | 0.94261 | 1.02 | 32799 | 1105 | 0.86226 |
| rs2823139 | 21 | 16576783 | A | G | 0.33 | **1.06** | **45191** | **4150** | **0.02048** | 0.98 | 32799 | 1105 | 0.65373 |
| rs2834317 | 21 | 35356706 | A | G | 0.11 | 1.02 | 45191 | 4150 | 0.70447 | 1.15 | 32799 | 1105 | 0.12468 |
| rs17001977 | 22 | 40880213 | G | A | 0.93 | 0.96 | 45191 | 4150 | 0.42132 | 1.17 | 32799 | 1105 | 0.09796 |

Supplementary Figure 1. Quantile-quantile plots for CKD (A) and ESKD (B)

A. B.


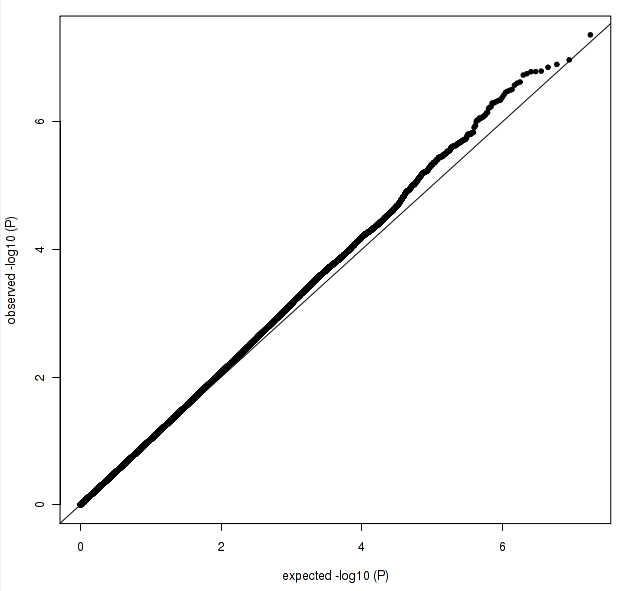

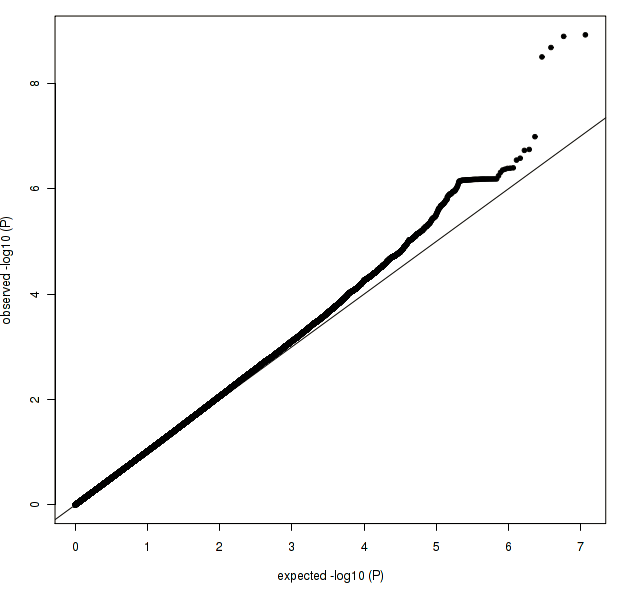

Supplement: Supplementary file 1 [file Table_1.docx]
